# 

**Published:** 1897-01-09

**Authors:** 


### EARLY EDITION.

**Notices of Births, Deaths, and Marriages** are inserted in this column at a charge of 2s. 6d. for an announcement not exceeding four lines.

### NOTICE TO CORRESPONDENTS.

All MS., Letters, Books for Review, and other matters intended for the Editor should be addressed THE EDITOR, "THE HOSPITAL," THE HOSPITAL BUILDING, 28 & 29, SOUTHAMPTON STREET, STRAND, W.C.

The Editor cannot undertake to return rejected MS., even when accompanied by stamped directed envelope.

**Notice to Advertisers and Subscribers.**

All Advertisements, Orders for Copies of THE HOSPITAL, and Business Communications should be addressed to **THE MANAGER, (Not to The Editor),** THE HOSPITAL, The Hospital Building, 28 & 29, Southampton Street, Strand, London, W.C.

The Cost of Subscription, post free, is as follows:—Per week, 2½d.; per quarter, 2s. 8d.; per half-year, 5s. 3d.; per annum, 10s. 6d. Foreign:—Per annum, 15s. 2d.; payable, in all cases, in advance.

**NOTICE.—Vol. XX. of "The Hospital" (April to September, 1896), in handsome cloth binding, is now on sale at the Office of this Journal, price 7s. 6d. Cases for binding the Numbers contained in this Volume. 1s. 6d. Index to Vol. XX., 2½d., post free. The Monthly Part for January is now ready. Price 6d., by post 8d.**

### SCRAPS AND GLEANINGS.

CORK Hospital Saturday collection realised £490 in 1896, as against £570 in 1895.

IT is proposed to build an operation ward in connection with the Children's Cottage Hospital, Cold Ash.

SEVERAL letters have appeared in the Manchester papers advocating the establishment of a temperance hospital for that town.

MANCHESTER Hospital Sunday and Saturday Fund collection for 1896 shows a slight increase over that for 1895, the amounts being £8,315 and £8,200 respectively.

JUDGING from the speeches made at a recent meeting of the Committee of the Stockton Surgical Hospital, the lot of the officials of late has been anything but a happy one.

A NEW accident hospital is about to be erected at Sunnyside, Alloa, by Miss Forrester Paton. It is to be handed over to the committee of the present hospital when finished.

THE Committee of Queen Charlotte's Lying-in Hospital, Marylebone, have received a donation of £50 from the executor of the late Sir Albert Sassoon in aid of the Extension Fund.

FOUR HUNDRED AND THIRTY-SEVEN patients were received into the wards of the Cork Eye, Ear, and Throat Hospital during the year 1895-6, while 4,062 were treated in the extern department.

THE Coventry and Warwickshire Hospital has treated 616 in and 5,693 out patients during the year 1895-6. The expenditure exceeded the income by about £440, £3,374 having been received and £3,815 expended.

MR. J. ST. S. WILDERS, who for nearly forty years has been on the staff of the Queen's Hospital, Birmingham, has resigned his position as an acting surgeon, and has been appointed consulting surgeon to the hospital.

THE number of patients attended at the Manchester Ear Hospital last year shows a large increase over that of the previous year, the figures being 102 in and 1,849 out in 1895-6, against 60 in and 1,638 out in 1894-5.

THE report of the Brixham Cottage Hospital shows that the hospital has an adverse balance of £25. Sixty-seven in-patients have been treated during the past year, and 674 patients have been visited by the district nurse.

THE Southport Infirmary have issued a circular regretting the attempt which has been made to establish an Eye, Ear, and Throat Hospital at Southport, it being contended that suitable provision for this class of cases has been made at the infirmary.

THE board of the Northern Counties Hospital for Incurables, Mauldeth, undaunted by the fact that the appeal they issued for funds for this institution met with a most disappointing response, have instituted a system of pensions for incurables which will come into force in May next.

ONE THOUSAND SIX HUNDRED AND NINE patients have received treatment at the Victoria Hospital, Glasgow, during the year 1895-6, as against 1,494 during the previous year. In addition, 245 slight cases of accident were treated. At the outdoor dispensary 11,417 consultations took place, as compared with 9,326 the previous year. The ordinary income amounted to £6,238 and the ordinary expenditure to £7,162. The extraordinary income amounted to £5,641.

THE report of the North Staffordshire Infirmary for the year ended October, 1896, shows that the income from all sources has been £10,325, as compared with £12,120 in the previous year, or a decrease of £1,795, while there has been a decreased expenditure of only £15. The deficiency has been largely caused by the falling off in donations and legacies. 11,779 persons (1,957 in and 9,822 out-patients) underwent treatment at the infirmary, 24 fewer than in the preceding year, but considerably over the average for the past five years.

THE Royal Infirmary, Edinburgh, received during the year just closed £30,683 from ordinary sources and £76,364 from legacies and donations over £100. Of the latter sum £50,000 was bequeathed by the late Earl of Moray and £11,000 by the late Mr. Peter Waddell, of Leith. The ordinary expenditure amounted to £39,282. During the past six years the number of out-patients has increased from 23,000 to 28,000, and the in-patients from 8,769 to 9,478, with a daily average number of beds occupied of 648 and 706 respectively.

THE number of patients treated at the dispensary in connection with the Western Infirmary, Glasgow, during the year ended October 31st, 1896 (14,136), is more than that of the preceding year by 409, while the indoor cases treated (4,542) show a decrease of one for the same period. The average daily number of indoor patients was 385, as compared with 392 in the previous year. The greatest number of patients in the hospital at one time was 425, and the smallest number was 332. The average period of residence of each patient was 31·02 days, against 31·47 last year.

### Books Received.

Sampson Low, Marston, and Co.

"Twentieth Century Practice of Modern Medical Science," vol. vii.

Bailliere, Tindall, and Cox.

"Cycling as a Cause of Heart Disease." By George Herschell, M.D.

Burns and Oates.

"The Catholic Directory for 1897."

The Vegetarian Federal Union.

"The Vegetarian Year Book, 1897."

The Roxburghe Press.

"The Rosy Cross." By Mina Sandeman.

**Periodicals, &c.** — Girls' Own Paper, Boys' Own Paper, Leisure Hour, Sunday at Home, Sunday Hour, Industries and Iron, Medical World, London, St. Bartholomew's Hospital Journal, Cassell's Family Magazine, Review of Reviews, Gentleman's Journal, Cornhill Magazine, Literary Digest, Electrical Review, British Review, Appleton's Popular Science, Charities Review, Westminster Review, The Gardeners' Magazine, Gardening Year Book, Whitaker's Almanack, 1897.

### The Student of Medicine.

### APPOINTMENTS.

E. W. Adams, F.R.C.S.Eng., L.R.C.P.Lond., D.P.H., appointed Medical Officer of Health to the Slough Urban District Council. W. L. Bentley, L.S.A., appointed Medical Officer for the No. 2 District of the Oldham Union. T. Bryant, F.R.C.S., appointed Consulting Surgeon to the Hospital for Epilepsy and Paralysis, and other Diseases of the Nervous System, Regent's Park. H. S. Cooper, M.R.C.S., L.R.C.P.Lond., L.S.A., appointed Medical Officer of Health to the Brightlingsea Urban District Council. A. R. S. Freeland, M.R.C.S.Eng., L.R.C.P.Lond., appointed Resident Medical Officer to the Barnes Convalescent Hospital, Cheadle, Cheshire. A. C. Fry, M.A., L.R.C.P., M.R.C.S., appointed House Surgeon to the Royal Berkshire Hospital, Reading. W. A. Gibb, M.B., C.M.Edin., appointed Junior House Surgeon to the General Infirmary, Gloucester. G. R. Gowland, L.R.C.P., L.R.C.S.Edin., appointed Medical Officer for the No. 7 District of the Chorlton Union. J. C. Keer, M.R.C.S.Eng., L.S.A., appointed Medical Officer of the Workhouse and of the Wickham Market District of the Plomesgate Union. Mr. McAnally, appointed Medical Officer for the Workhouse and Eastry District of the Eastry Union. A. H. W. Macdonald, M.B., C.M.Edin., appointed Medical Officer for the Old Ford District of the Poplar Union. W. G. R. Macauley, L.R.C.P., M.R.C.S., L.S.A., appointed Medical Officer to the Freebridge Lynn Union, Government Certifying Factory Surgeon, Medical Officer Court of Oddfellows, and other Clubs, King's Lynn. J. Spurr, B.A.Camb., M.R.C.S.Eng., appointed Medical Officer of Health to the Borough of Lyme Regis. J. D. O. Wilson, M.B., C.M.Glas., appointed House Surgeon to the Newcastle-on-Tyne Hospital for Sick Children (Fleming Memorial). J. B. Yelf, M.R.C.S., L.R.C.P.Lond., appointed Medical Officer for the Shipston-on-Stour District of the Shipston-on-Stour Union. Dr. Young, appointed Medical Officer for the Hawkhurst District of the Cranbrook Union.

### ROYAL NATIONAL PENSION FUND FOR NURSES.

*President*—H.R.H. THE PRINCESS OF WALES.

*Chairman*—WALTER H. BURNS, Esq. *Deputy-Chairman*—HENRY C. BURDETT, Esq.

PENSIONS. SICKNESS. ACCIDENT.

### Total Invested Funds, £300,000.

Nurses are invited to join the Fund on account of the substantial and exceptional advantages which it offers them and which *they cannot obtain elsewhere.* The following are the chief points:—

1. **The Fund is Mutual and essentially Co-operative.**
2. **Easy Payment of Premiums.**
   Nurses can pay their premiums monthly or otherwise as best suits their convenience.
3. **The Fund is open to every Nurse.**
   Nurses can assure for Pensions of any amount commencing at any age.
4. **An Investment and Savings Bank.**
   Those entering under the returnable scale can have their premiums returned to them with compound interest, less a small deduction for working expenses. Interest allowed on deposits.
5. **Additions to Pensions.**
   Besides the Pension entered for, substantial additions thereto may be anticipated in the shape of bonuses.
6. **Sickness and Accident Assurance.**
   Policies are issued in connection with Pension policies assuring 10s., 15s., or 20s. a week in cases of incapacity from work through sickness or accident.

Full particulars to be obtained from THE SECRETARY,

ROYAL NATIONAL PENSION FUND FOR NURSES,

28, FINSBURY PAVEMENT, LONDON, E.C

